# In-Hospital Outcomes in Patients With Non-ST Segment Elevation Myocardial Infarction and Concomitant Neurodevelopmental Disorders in the United States: Insights From the National Inpatient Sample 2011-2020

**DOI:** 10.7759/cureus.60289

**Published:** 2024-05-14

**Authors:** Ian Ergui, Nayrana Griffith, Joshua Salama, Bertrand Ebner, Michael Dangl, Louis Vincent, Victor Razuk, George Marzouka, Rosario Colombo

**Affiliations:** 1 Internal Medicine, University of Miami Miller School of Medicine/Jackson Memorial Hospital, Miami, USA; 2 Cardiology, University of Miami Miller School of Medicine/Jackson Memorial Hospital, Miami, USA; 3 Cardiology, Miami Department of Veterans Affairs, Miami, USA

**Keywords:** acute coronary syndrome, healthcare equity, neurodivergent, primary percutaneous coronary intervention (pci), non-st segment elevation myocardial infarction (nstemi)

## Abstract

Patients with neurodevelopmental disorders (NDDs) encounter significant barriers to receiving quality health care, particularly for acute conditions such as non-ST segment elevation myocardial infarction (NSTEMI). This study addresses the critical gap in knowledge regarding in-hospital outcomes and the use of invasive therapies in this demographic. By analyzing data from the National Inpatient Sample database from 2011 to 2020 using the International Classification of Diseases, Ninth Edition (ICD-9) and Tenth Edition (ICD-10) codes, we identified patients with NSTEMI, both with and without NDDs, and compared baseline characteristics, in-hospital outcomes, and the application of invasive treatments. The analysis involved a weighted sample of 7,482,216 NSTEMI hospitalizations, of which 30,168 (0.40%) patients had NDDs. There were significantly higher comorbidity-adjusted odds of in-hospital mortality, cardiac arrest, endotracheal intubation, infectious complications, ventricular arrhythmias, and restraint use among the NDD cohort. Conversely, this group exhibited lower adjusted odds of undergoing left heart catheterization, percutaneous coronary intervention, or coronary artery bypass graft surgery. These findings underscore the disparities faced by patients with NDDs in accessing invasive cardiac interventions, highlighting the need for further research to address these barriers and improve care quality for this vulnerable population.

## Introduction

In the United States, recent data highlight that nearly 18% of the population is affected by neurodevelopmental disorders (NDDs), a 9.5% increase over the past decade [1]. Classified by the Diagnostic and Statistical Manual of Mental Disorders, 5th Edition (DSM-V) as conditions manifesting early in life that lead to enduring developmental deficits impacting occupational, academic, and social functioning, NDDs encompass a spectrum of conditions, including autism spectrum disorder (ASD), attention-deficit/hyperactivity disorder (ADHD), intellectual disability (ID), specific learning disorders (LD), motor disorders (MD), and communication disorders (CD) [2]. Often co-occurring, these disorders increase the susceptibility to cardiovascular diseases such as atherosclerosis, dyslipidemia, and heart failure [3-7].

While the connection between NDDs and heightened cardiovascular disease risk remains underexplored, it is suspected to be multifactorial, involving lifestyle factors, medication effects, genetic predispositions, and prenatal exposures [3,6-8]. Genetic studies in mice have begun to link NDD-related genetic variants to cardiac abnormalities, suggesting a complex interplay between neurological and cardiovascular health [9-29].

Despite the well-documented prevalence of NDDs [30-34] and the associated cardiovascular risk, there is a notable scarcity of data on the in-hospital outcomes of patients with NDDs and non-ST segment elevation myocardial infarction (NSTEMI). Previous studies indicate a significant disparity in the utilization of invasive therapies for these patients, coupled with poorer short and long-term outcomes [35,36]. This gap in care, potentially exacerbated by interpersonal and institutional biases [37], underscores the urgent need for research into the in-hospital outcomes of NSTEMI patients with NDDs, hypothesizing that these patients face higher mortality rates and reduced access to invasive treatments compared to their neurotypical counterparts.

## Materials and methods

Study design

This study was a retrospective analysis that leveraged data from the National Inpatient Sample (NIS), the most extensive all-payer inpatient care database in the United States. The NIS is managed by the Healthcare Cost and Utilization Project (HCUP), which is sponsored by the Agency for Healthcare and Research Quality (AHRQ). The NIS database, which does not include long-term acute care and rehabilitation hospitals, offers anonymized patient, hospital, and state-level data, obviating the need for Institutional Review Board approval due to the de-identification of patient information. As per NIS recommendations, all analyzed data were weighted to ensure representativeness.

**Figure 1:**
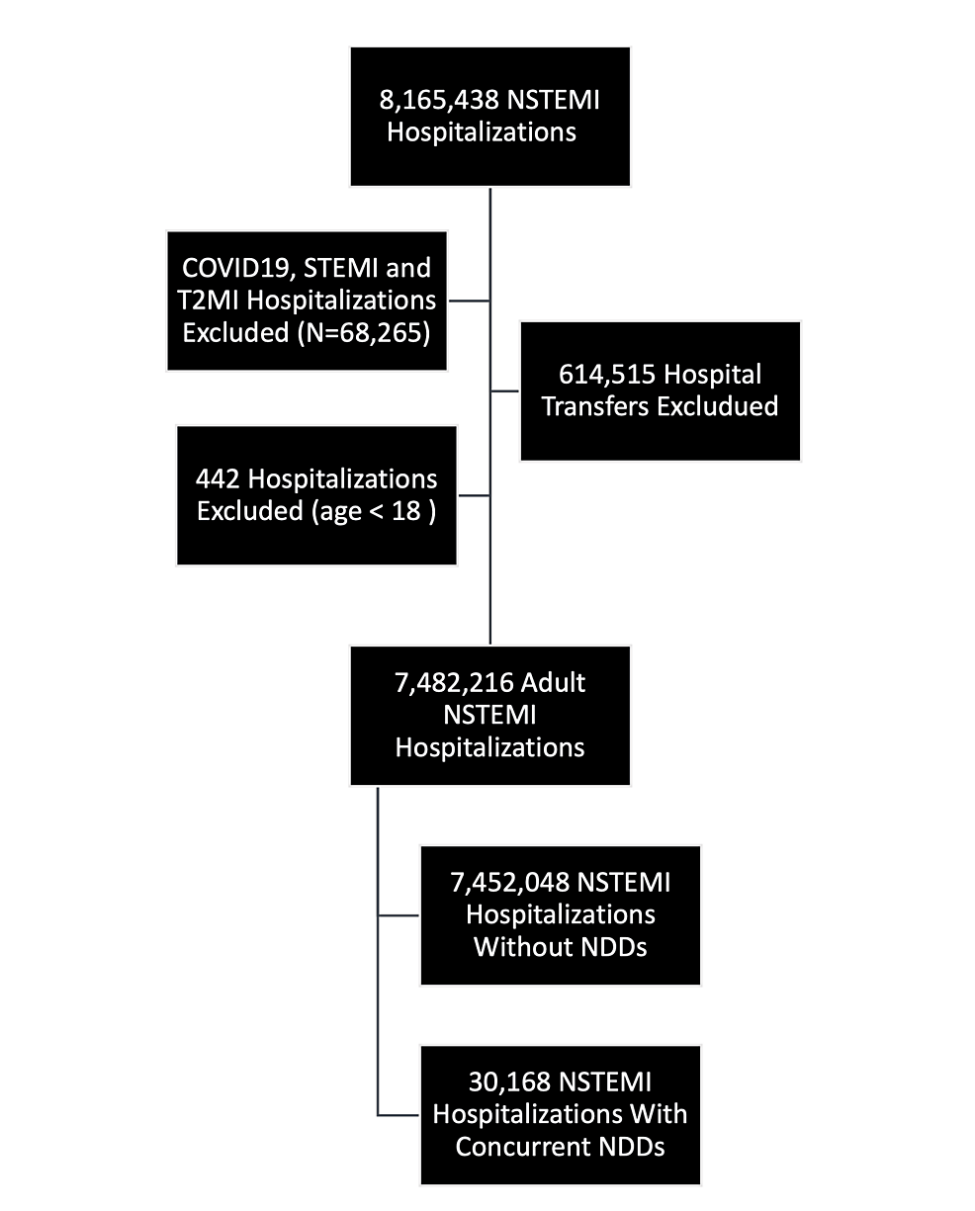
Flowchart detailing the selection of NSTEMI hospitalizations between 2011 and 2020 with and without NDDs. STEMI: ST-segment elevation myocardial infarction; NSTEMI: non-ST segment elevation myocardial infarction; T2MI: type 2 myocardial infarction; NDDs: neurodevelopmental disorders.

Between January 1, 2011, and December 31, 2020, the NIS was queried for all inpatient hospitalizations, resulting in 356,578,336 weighted cases. NSTEMI hospitalizations were isolated using specific International Classification of Diseases, Ninth Edition (ICD-9) and Tenth Edition (ICD-10) codes (Supplemental Material Table A1), resulting in 8,165,438 identified cases. Exclusions were made for patients under 18 years, those transferred out of the hospital, patients with COVID-19, and cases identified as ST-segment elevation myocardial infarction (STEMI) or type 2 myocardial infarctions, culminating in a cohort of 7,482,216 NSTEMI hospitalizations (Figure 1). Of the total NSTEMI population, approximately 8.5% were from the year 2011 (633,216), 8.5% from 2012 (638,735), 8.6% from 2013 (641,615), 8.7% from 2014 (604,370), 9.2% from 2015 (685,295), 9.4% from 2016 (699,915), 10.7% from 2017 (798,435), 11.8% from 2018 (880,835), 12.6% from 2019 (939,770), and 12.2% from 2020 (910,010). Within this cohort, 30,168 patients (0.40%) were documented to have NDDs (Supplemental Material Table A2). Of the patients with NDDs, approximately 50.50% had ID (n = 15,223), 28.71% had ADHD (n = 8,470), 3.28% had CDs (n = 989), 2.07% had specific learning disorders (n = 625), 7.60% had ASD (n = 2,290), and 2.74% had MDs (n = 827).

Baseline characteristics such as age, gender, non-white race, elective admission status, and cardiovascular comorbidities were systematically assessed. The primary endpoint was in-hospital mortality, with secondary outcomes, including the incidence of percutaneous coronary intervention (PCI), coronary artery bypass grafting (CABG), and various complications, including cardiac arrest, stroke, infections, and major bleeding (Supplemental Material Table A3).

For data analysis, descriptive statistics were applied to both continuous and categorical variables, with missing data addressed through multiple imputations, as recommended by HCUP. Continuous variables were analyzed using appropriate tests based on their distribution, and categorical variables were assessed with Pearson’s chi-square test. Statistical significance was set at p-values < 0.05. Binary logistic regression facilitated the calculation of adjusted odds ratios (aORs) for in-hospital outcomes, adjusting for variables with significant baseline differences. The statistical software SPSS (IBM SPSS Statistics for MAC, version 28.0; IBM Corp., Armonk, NY) supported these analyses, with regression models for a comprehensive understanding of the impact of NDDs on NSTEMI hospitalizations.

## Results

The baseline characteristics of NSTEMI patients with and without NDDs, detailed in Table 1, reveal that neurotypical patients were generally older and more frequently identified as female, with a higher incidence of elective admissions.

**Table 1:** Baseline characteristics of patients with and without NDDs admitted with NSTEMI. Values are reported as mean ± standard deviation for continuous variables and percentage (number) for categorical variables. * P-value ≤ 0.05 is considered significant. NSTEMI: non-ST segment elevation myocardial infarction; NDDs: neurodevelopmental disorders.

Variable	No NDD (n = 7,452,048)	NDD (n = 30,168)	p-value
Age	70.06 ± 13.82	59.03 ± 15.15	<0.05*
Female	43.9% (3,269,585)	38.8% (11,706)	<0.05*
Non-white race	27.0% (2,014,319)	20.4% (6,152)	<0.05*
Elective admission	6.2% (458,013)	4.9% (1,488)	<0.05*
Hypertension	84.9% (5,967,009)	71.0% (20,099)	<0.05*
Diabetes mellitus	42.2% (3,125,930)	34.8% (10,322)	<0.05*
HIV/AIDS	0.4% (33,443)	0.7% (214)	<0.05*
Chronic kidney disease	31.9% (2,369,757)	21.7% (6,484)	<0.05*
On hemodialysis	4.0% (301,324)	2.8% (858)	<0.05*
History of heart failure	48.3% (3,599,755)	41.6% (12,555)	<0.05*
Prior stroke	11.8% (877,456)	8.6% (2,607)	<0.05*
Tobacco use	40.0% (2,977,676)	32.7% (9,869)	<0.05*
Alcohol use	3.9% (291,769)	4.7% (1,402)	<0.05*
Drug use	3.4% (249,671)	8.6% (2,575)	<0.05*
Anemia	6.7% (417,358)	7.1% (1,890)	<0.05*

In this analysis, a minority of patients were categorized as elective admissions yet developed NSTEMI subsequently during their hospital stay. Specifically, 6.2% (458,013) of patients in the neurotypical group and 4.9% (1,488) in the NDD group were admitted electively. These admissions generally pertained to individuals scheduled for non-emergent procedures or evaluations. Chronic conditions such as hypertension, diabetes, and a history of heart failure were more prevalent among the neurotypical cohort, while the NDD group presented more commonly with HIV/AIDS, substance abuse disorders, and anemia.

Notably, Table 2 illustrates that unadjusted in-hospital outcomes for patients with NDDs included higher mortality rates and increased incidences of endotracheal intubation and infectious complications, contrasted with the neurotypical group, which had higher frequencies of invasive cardiac procedures. Despite this, neurotypical patients experienced shorter hospital stays, with no significant difference in costs compared to the NDD group.

**Table 2:** Events and outcomes for NSTEMI hospitalizations. Outcomes reported as median (interquartile range) for non-parametric continuous variables and percentage (number) for categorical variables. ^a^ Infectious complications include all-cause infection, sepsis, or septic shock. ^b^ Length of stay reported in days. ^c^ Cost of stay reported in US dollars. * P-value < 0.05 was considered significant. NSTEMI: non-ST segment elevation myocardial infarction; NDDs: neurodevelopmental disorders; PCI: percutaneous coronary intervention; CABG: coronary artery bypass grafting.

Variable	No NDD (n = 7,452,048)	NDD (n = 30,168)	p-value
Mortality	8.0% (596,832)	9.0% (2,704)	<0.05*
PCI	24.6% (5,619,632)	16.1% (4,852)	<0.05*
CABG	3.4% (252,406)	1.3% (395)	<0.05*
Left heart cath	43.4% (3,231,110)	32.4% (9,762)	<0.05*
Cardiac arrest	3.1% (227,516)	4.7% (1,425)	<0.05*
Intubation	8.6% (641,772)	13.7% (4,137)	<0.05*
Stroke complications	4.9% (368,536)	4.1% (1239)	<0.05*
Infectious complications^a^	14.3% (1,066,630)	22.7% (6,836)	<0.05*
All major bleeding	8.6% (644,673)	7.6% (2,284)	<0.05*
Ventricular arrhythmia	1.3% (99,929)	1.7% (519)	<0.05*
Vascular complications	1.1% (79,006)	0.7% (225)	<0.05*
Restraint use	1.2% (89,665)	2.3% (696)	<0.05*
Length of stay^b^	6.37 (IQR 7.49)	7.34 (IQR 6)	<0.05*
Cost of stay^c^	$94,810 (IQR $78,688)	$91,695 (IQR $79,326)	0.063

**Figure 2:**
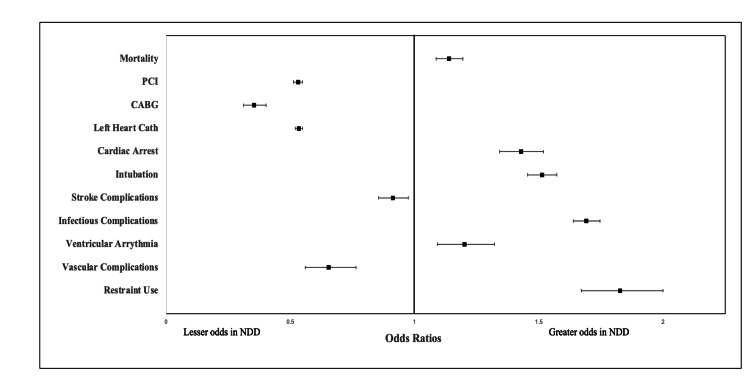
Adjusted in-hospital outcomes and events in neurotypical versus NDD patients with NSTEMI. Values > 1 indicate higher odds in patients with NDDs. NSTEMI: non-ST segment elevation myocardial infarction; NDDs: neurodevelopmental disorders; PCI: percutaneous coronary intervention; CABG: coronary artery bypass grafting.

Adjusted analysis using binary logistic regression (Figure 2) indicated that patients with NDDs had higher odds of in-hospital mortality (aOR: 1.138, 95% CI: 1.086-1.194, p < 0.05), ventricular arrhythmia (aOR: 1.201, 95% CI: 1.092-1.322, p < 0.05), and cardiac arrest (aOR: 1.428, 95% CI: 1.342-1.519, p < 0.05). Inversely, their likelihood of receiving invasive cardiac interventions such as left heart catheterization (aOR: 0.534, 95% CI: 0.519-0.549, p < 0.05), PCI (aOR: 0.531, 95% CI: 0.513-0.549, p < 0.05), or CABG (aOR: 0.354, 95% CI: 0.312-0.402, p < 0.05) was significantly lower. Regarding in-hospital complications, patients with NDDs had higher odds of infectious complications (aOR: 1.691, 95% CI: 1.638-1.746, p < 0.05), yet lower odds of stroke complications (aOR: 0.912, 95% CI: 0.854-0.974, p < 0.05) and vascular complications (aOR: 0.654, 95% CI: 0.560-0.764, p < 0.05). Despite fewer invasive procedures, the NDD group had higher odds of requiring endotracheal intubation (aOR: 1.513, 95% CI: 1.455-1.573, p < 0.05) and restraint use (aOR: 1.827, 95% CI: 1.670-1.999, p < 0.05).

## Discussion

In this cohort, we demonstrate that, overall, the presence of NDDs was associated with increased odds of in-hospital mortality, ventricular dysrhythmia, cardiac arrest, restraint use, and endotracheal intubation. This is despite the fact that patients with NDDs were younger and had fewer traditional cardiovascular comorbidities than their neurotypical counterparts. The observed increased odds of death or respiratory failure may be secondary to the decreased odds of undergoing cardiac catheterization, PCI, or CABG for NSTEMI patients with NDDs. Conversely, the increased odds of stroke and vascular complications in neurotypical patients may be secondary to the increased frequency of invasive procedures in that group or the overall older age and great prevalence of comorbid conditions in that population.

We present the first nationwide retrospective analysis of NSTEMI outcomes in adult patients with NDDs. While it is widely accepted that early invasive management of high-risk NSTEMI is associated with improved outcomes [38-40], neurodivergent patients face significant obstacles when admitted with occlusion myocardial infarction. A prior study of the Chest Pain - MI Registry showed that elderly patients with cognitive impairment are less likely to receive coronary angiography, PCI, and CABG [35]. Patients with cognitive impairments and NSTEMI had higher in-hospital mortality than neurotypical patients in that study (11.6% unadjusted mortality in cognitively impaired patients versus 3.5% mortality in neurotypical patients in the Chest Pain - MI Registry versus 9.3% unadjusted mortality in NDD patients and 8.0% mortality in neurotypical patients in this study); more severe cognitive impairments were associated with worse outcomes [35,41]. Patients with dementia are objectively less likely to undergo angiography or revascularization in the setting of acute myocardial infarction and have higher 30-day and one-year mortality compared to controls [42]. Dementia is a known barrier to care in patients with STEMI despite strict guidelines regarding time to reperfusion [43]. Older adults with cognitive impairment without dementia also had fewer percutaneous or surgical revascularization procedures and referrals to cardiac rehabilitation programs after acute myocardial infarction [41]. Prior major trials for revascularization in NSTEMI likely excluded patients with NDDs, dementia, and cognitive impairment. The TIMACS trial excluded patients deemed unsuitable for PCI, yet clear criteria for suitability were not offered [40]. While these exclusions likely mirror common practices in the community, they have consequently resulted in a lack of data on the benefits of revascularization for NSTEMI in patients with NDDs.

By definition, patients with neurodevelopmental disorders suffer from lifelong deficits that may hinder interpersonal communication [2]. Patients with disabilities that impair communication skills suffer from higher frequencies of preventable adverse events than their peers [44]. Patients with intellectual disabilities admitted for surgical emergencies present with more severe diseases and suffer from poorer outcomes [45]. With regards to patients presenting with NSTEMI, there may be discordance between symptoms expressed by patients with NDDs and the classical clinical presentation that healthcare providers are trained to expect [46]. Patients with NDDs may have delayed time to presentation in the hyperacute phase of myocardial infarction and present with a sicker phenotype [45,47].

Additionally, provider-level concerns regarding dual antiplatelet therapy adherence in NDD patients may be in part responsible for the lower odds of invasive therapy for NSTEMI seen in this study. A prior study of adults with intellectual disabilities and hypertension showed that about 50% were compliant with anti-hypertensive therapy [48]. Proceduralists may understandably be hesitant to proceed with revascularization given the presumed low rates of adherence in patients with NDDs; however, this generalized approach to the care of neurodivergent populations should be cautioned against. Not all patients with NDDs are at equal risk of medication non-adherence; patient-level characteristics such as preserved memory and executive functioning are associated with improved compliance [49]. Additionally, interventions such as structured living settings, automated short message reminders to take medications, and close contact with primary care providers have been shown to improve adherence in patients with NDDs [21,48]. These factors and others should be considered prior to making the decision to forego revascularization in patients with NDDs and NSTEMI.

Patients with NDDs have an increased risk for early-onset cardiovascular disease, and the severity of that disease has been correlated to the severity of disability [5]. Up to one-third of patients with cardiovascular disease may have some form of cognitive impairment [50]. Acquired neurocognitive disorders and cardiac disease share similar risk factors such as obesity, physical inactivity, smoking, depression, hypertension, diabetes mellitus, and socioeconomic disadvantage [50]. The long-term use of medication for ADHD is associated with an increased risk of coronary artery disease [51]. Risk factors such as obesity, hyperlipidemia, hypertension, and diabetes are common in autistic adults [52].

Societal guidelines and advances in healthcare delivery have revolutionized the care of patients with acute coronary syndromes [53]. The protocolized quantification of symptoms, cardiac biomarkers, patient history, and electrocardiographic findings has resulted in standardized scoring systems to determine which patients benefit from early invasive management of acute coronary syndrome [40,54]. Our study indicates that, despite the attempt to standardize care across groups, patients with NDDs are not being intervened on, and this may be the cause of the increased mortality and cardiopulmonary failure incidence seen in the dataset.

Patients with NSTEMI may present with a range of symptoms, from nausea and fatigue to refractory substernal chest pressure and hemodynamic instability requiring mechanical circulatory support. Patients with NDDs represent a vulnerable population that may not be able to communicate subjective symptoms, and healthcare systems should strive to close gaps in care that may be responsible for the poor clinical outcomes observed in this study. There are movements underway to recategorize patients with NDDs under the “neurodiversity approach”: a framework that rejects the normalization of any singular neural paradigm, promotes self-advocacy among patients with NDDs, and prioritizes adapting systems and environments to a neurologically diverse population [55,56]. Strong collaborations between healthcare systems and social services will be needed to actualize improvements in care for this population. Prioritizing primary care, mental health support, and income assistance are some potential areas of opportunity to bridge the observed disparities of care in this study.

The current study has several limitations. While patients with STEMI and type 2 myocardial infarctions were excluded from the analysis, the granularity of ICD-9 and ICD-10 codes makes assessing the severity of the NSTEMI by objective factors such as cardiac biomarkers and EKG findings impossible. Patient level descriptors such as symptoms, vital signs, and laboratory findings such as high sensitivity troponin were not available for review. There is a lower frequency of coronary angiography for patients with NSTEMI than would be expected. The NIS, which records data at discharge, may not accurately reflect procedures like coronary angiography in patients that were transferred, potentially leading to underreporting. While hospital transfers were expressly excluded from the analysis, the potential for facility-level miscoding exists. Additionally, variability in clinical practice across the US, affected by regional disparities, resource constraints, and adherence to evolving clinical guidelines, can influence the frequency of angiography. This is especially true in rural or underserved hospitals and during periods of changing practice patterns, possibly contributing to the lower observed rates in our dataset. For the patients who underwent angiography, information regarding coronary anatomy and culprit lesion complexity is not available. Echocardiographic readings and other predictors of poor outcomes are not offered. The relatively low sample size of NDD patients ensures that any observations drawn from the data are primarily descriptive and causation cannot be inferred. Further studies are needed to better characterize the ACS severity at presentation for patients with and without NDDs along with post-discharge outcomes.

The disparity between the overall increased incidence of traditional cardiac comorbidities seen in the neurotypical population in this study, the decreased implementation of aggressive NSTEMI therapy, and the increased odds of death in the NDD population show a clear gap that healthcare providers and systems must bridge to ensure equal care for these vulnerable patients. Patients with NDDs and their carers consistently express feelings of direct and indirect discrimination from health service providers [57]. There is ongoing research regarding reasonable adjustments that hospital systems can implement to improve the care of patients with NDDs. Access to specialized liaison nurses and staff, additional resources, and national guidance have been shown to improve outcomes for patients with NDDs [58]. Heart teams should consider a multidisciplinary approach to caring for patients with NDDs and NSTEMI to ensure that patients who cannot advocate for themselves are treated equally to those who can.

## Conclusions

Patients with NDDs and NSTEMI have higher overall mortality, yet lower implementation of revascularization therapies compared to neurotypical patients despite being an overall younger and healthier cohort. When adjusted for comorbidities, NDD status was independently associated with an increased likelihood of death, endotracheal intubation, cardiac arrest, and ventricular dysrhythmia. Further studies are needed to develop advancements in current healthcare delivery models to address these disparities in care.

## Appendices

Supplemental material

The NIS contains data on inpatient hospitalizations from participating states (n = 47 and the District of Columbia). This is the largest all-payers inpatient dataset, approximating a 20% sample of US hospitalizations from community hospitals (defined as general or subspecialty short-term hospitals, excluding rehabilitation and long-term acute care facilities). Over 1000 hospitals contribute data from about 7 million discharges annually. The NIS approximates more than 97% of the US population. Patient, hospital, and state-level identifiers are excluded from the database. Institutional review board approval was unnecessary as all information in the NIS is de-identified. Discharge weights are provided with all records to derive national estimates. All data were weighted as recommended by the NIS to derive national estimates. Prior to October 1, 2015, hospital administrative data in the US were encoded using the International Classification of Diseases, Ninth Edition, Clinical Modification/Procedure Coding System (ICD-9-CM/PCS). The last three months of 2015, and from 2016 onwards, the NIS contained data based on the International Classification of Diseases, Tenth Edition, Clinical Modification/Procedure Coding System (ICD-10-CM/PCS) codes. These changes are accounted for in the substructure of the NIS.

Table A1

**Table 3:** Codes used to identify NSTEMI hospitalizations. NSTEMI: non-ST segment elevation myocardial infarction; ICD: International Classification of Diseases.

Variable	ICD 9 and 10 codes
NSTEMI	I214, I21A1, I222, 41071, 41070, 41072

Table A2

**Table 4:** Diagnostic codes used to identify patients with neurodevelopmental disorders. ICD: International Classification of Diseases.

ICD 10 codes	Code description	ICD 10 codes	Code description
'F840'	Autistic disorder	'F812'	Mathematics disorder
'F842'	Rett's syndrome	'F8181'	Disorder of written expression
'F843'	Other childhood disintegrative disorder	'F8189'	Other developmental disorders of scholastic skills
'F845'	Asperger's syndrome	'F819'	Developmental disorder of scholastic skills, unspecified
'F848'	Other pervasive developmental disorders	'F82'	Specific developmental disorder of motor function
'F849'	Pervasive developmental disorder, unspecified	'F88'	Other disorders of psychological development
'F70'	Mild intellectual disabilities	'F89'	Unspecified disorder of psychological development
'F71'	Moderate intellectual disabilities	'F900'	Attention-deficit hyperactivity disorder, predominantly inattentive type
'F72'	Severe intellectual disabilities	'F901'	Attention-deficit hyperactivity disorder, predominantly hyperactive type
'F73'	Profound intellectual disabilities	'F902'	Attention-deficit hyperactivity disorder, combined type
'F78'	Other intellectual disabilities	'F908'	Attention-deficit hyperactivity disorder, other type
'F78A1'	SYNGAP1-related intellectual disability	'F909'	Attention-deficit hyperactivity disorder, unspecified type
'F78A9'	Other genetic-related intellectual disability	'F948'	Other childhood disorders of social functioning
'F79'	Unspecified intellectual disabilities	'F949'	Childhood disorder of social functioning, unspecified
'F800'	Phonological disorder	'F950'	Transient tic disorder
'F801'	Expressive language disorder	'F951'	Chronic motor or vocal tic disorder
'F802'	Mixed receptive-expressive language disorder	'F952'	Tourette's disorder
'F809'	Developmental disorder of speech and language, unspecified	'F958'	Other tic disorders
'F8081'	Childhood-onset fluency disorder	'F959'	Tic disorder, unspecified
'F8082'	Social pragmatic communication disorder	'F984'	Stereotyped movement disorders
'F8089'	Other developmental disorders of speech and language	'F985'	Adult onset fluency disorder
'F810'	Specific reading disorder	'F988'	Other specified behavioral and emotional disorders with onset usually occurring in childhood and adolescence

Table A3

**Table 5:** ICD-9-CM/PCS and ICD-10-CM/PCS codes for comorbidities and NSTEMI complications. ICD-9-CM/PCS: International Classification of Diseases, Ninth Edition, Clinical Modification/Procedure Coding System; ICD-10-CM/PCS: International Classification of Diseases, Tenth Edition, Clinical Modification/Procedure Coding System; NSTEMI: non-ST segment elevation myocardial infarction; CABG: coronary artery bypass grafting.

Variables	ICD-9 and ICD-10 codes
COVID-19	U071, J1282, J1281
Hypertension	401.X, 402.X, 405.X, I10, I11.X, I12.X, I15.X
Diabetes mellitus	250.X, E08.X, E09.X, E10.X, E11.X, E12.X, E13.X, E14.X
HIV/AIDS	R75, B9735, Z21, 042, 07953, V08, 7957, B24
Chronic kidney disease	585.X, N18.X
Renal replacement therapy with hemodialysis	39.95. 5A1D00Z, 5A1D60Z, 5A1D70Z 5A1D80Z, 5A1D90Z
History of heart failure	40201, 40211, 40291, 40401, 40403, 40411, 40413, 40491, 40493, I110, I130, I132, 39891, 4254, 4255, 4256, 4257, 4258, 4259, I099, I255, I420, I425, I426, I427, I428, I429, P290, I43
Stroke history	438.X, I69.X, V12.54, Z86.73
Tobacco	305.1, 989.84, V15.82, F17.X, T65.2X, Z72.0, Z87.891
Alcohol	291.1, 291.2, 291.3, 291.5, 291.6, 291.7, 291.81, 291.89, 291.9, 265.2, 303.00, 303.90, 303.91, 303.92, 303.93, 305.00, 305.01, 305.02, 305.03, 357.5, 425.5, 535.3, 571.0, 571.1, 571.2, 571.3, 980.X, E52, F10.X, G62.1, I42.6, K29.2, K70.0, K70.3, K70.9, T51.X, V11.3, Z50.2, Z71.4, Z72.1
Substance abuse	292.X, 304.X, 305.2, 305.3, 305.4, 305.5, 305.6, 305.7, 305.8, 305.9, F11.X, F12.X, F13.X, F14.X, F15.X, F16.X, F18.X, F19.X, V65.42, Z71.5, Z72.2
Anemia	280.X, 281.X, D50.X, D51.X, D52.X, D53.X
Cardiac arrest	4275, I46.X
Stroke complications	430, I60.X, 431, 432.9, I61.X, 432.X, I62.X, 433.X, 434.X, 435.X, 436, 432.9, I63.X
Infectious complications	038.X, 995.91, 995.92, A40.X, A41.X, R65.2, 785.52, 998.02, T81.12, 998.51, 998.59, T814XXA, 996.60, 996.61, T826XXA
All major bleeding	998.11, 998.12, I97.621, I97.630, I97.631, I97.638, I97.410, I97.411, I97.418, I97.42, I97.410, I97.411, I97.418, I97.42, I97.610, I97.611, I97.618, I97.620, 568.81, K66.1, 456.0, 456.20, 530.21, 530.7, 530.82, 531.00, 531.01, 531.20, 531.21, 531.40, 531.41, 531.60, 531.61, 532.00, 532.01, 532.20, 532.31, 532.40, 532.41, 532.60, 532.61, 533.00, 533.01, 533.20, 533.21, 533.40, 533.41, 533.60, 533.61, 534.00, 534.01, 534.20, 534.21, 534.40, 534.41, 534.60, 534.61, 535.01, 535.11, 535.21, 535.31, 535.41, 535.51, 535.61, 535.71, 537.83, 537.84, 562.02, 562.03, 562.12, 562.13, 569.3, 569.85, 569.86, 578.X, I85.01, I85.11, K22.11, K22.6, K22.8, K25.0, K25.2, K25.4, K25.6, K26.0, K26.2, K26.4, K26.6, K27.0, K27.2, K27.4, K27.6, K28.0, K28.2, K28.4, K28.6, K29.01, K29.21, K29.31, K29.41, K29.51, K29.61, K29.71, K29.81, K29.91, K31.811, K57.11, K57.13, K57.31, K57.33, K62.5, K55.21, K31.82, K63.81, K92.0, K92.1, K92.2, 596.7, 599.70, 599.71, N32.89, R31.9, R31.0, 786.30, 786.39, R04.2, R04.9, 784.7, R04.0, 459.0, R58
Vascular complications	9982, 9992, 99771, 99772, 99779, 4470, I9751, I9752, T801XXA, T81710A, T81711A, T81718A, T8172XA, I770, 3956, 3931, 3941, 3949, 3952, 3957, 3959, 3979, 02QP0ZZ, 02QP3ZZ, 02QP4ZZ, 02QQ0ZZ, 02QQ3ZZ, 02QQ4ZZ, 02QR0ZZ, 02QR3ZZ, 02QR4ZZ, 02QW0ZZ, 02QW3ZZ, 02QW4ZZ, 02QX0ZZ, 02QX3ZZ, 02QX4ZZ, 02QS0ZZ, 02QS3ZZ, 02QS4ZZ, 02QV0ZZ, 02QV3ZZ, 02QV4ZZ, 900.X, 901.X, 902.X, 903.X, 904.X, 03Q.X, 04Q.X, 0W3.X, 02UP.X, 02UQ.X, 02U3.X, 02UR.X, 02US.X, 02UT,X, 02UV.X, 02UW.X, 03U.X, 03U.X
Left heart catheterization	B210010, B2100ZZ, B210110, B2101ZZ, B210Y10, B210YZZ, B211010, B2110ZZ, B211110, B2111ZZ, B211Y10, B211YZZ, B212010, B2120ZZ, B212110, B2121ZZ, B212Y10, B212YZZ, B213010, B2130ZZ, B213110, B2131ZZ, B213Y10, B213YZZ, B2150ZZ, B2151ZZ, B215YZZ, B2160ZZ, B2161ZZ, B216YZZ, B2170ZZ, B2171ZZ, B217YZZ, B2180ZZ, B2181ZZ, B218YZZ, B21F0ZZ, B21F1ZZ, B21FYZZ, 8850, 8853, 8854, 8855, 8856, 8857, 8859, 8858
Percutaneous coronary intervention	3606, 3607, 3609, 0066, 1755, 02703ZZ, 02704ZZ, 02713ZZ, 02714ZZ, 02723ZZ, 02724ZZ, 02733ZZ, 02734ZZ, 02Q03ZZ, 02Q04ZZ, 02Q13ZZ, 02Q14ZZ, 02Q23ZZ, 02Q24ZZ, 02Q33ZZ, 02Q34ZZ, 0270346, 027034Z, 0270356, 027035Z, 0270366, 027036Z, 0270376, 027037Z, 02703D6, 02703DZ, 02703E6, 02703EZ, 02703F6, 02703FZ, 02703G6, 02703GZ, 02703T6, 02703TZ, 02703Z6, 0270446, 027044Z, 0270456, 027045Z, 0270466, 027046Z, 0270476, 027047Z, 02704D6, 02704DZ, 02704E6, 02704EZ, 02704F6, 02704FZ, 02704G6, 02704GZ, 02704T6, 02704TZ, 02704Z6, 0271346, 027134Z, 0271356, 027135Z, 0271366, 027136Z, 0271376, 027137Z, 02713D6, 02713DZ, 02713E6, 02713EZ, 02713F6, 02713FZ, 02713G6, 02713GZ, 02713T6, 02713TZ, 02713Z6, 0271446, 027144Z, 0271456, 027145Z, 0271466, 027146Z, 0271476, 027147Z, 02714D6, 02714DZ, 02714E6, 02714EZ, 02714F6, 02714FZ, 02714G6, 02714GZ, 02714T6, 02714TZ, 02714Z6, 02714ZZ, 0272346, 027234Z, 0272356, 027235Z, 0272366, 027236Z, 0272376, 027237Z, 02723D6, 02723DZ, 02723E6, 02723EZ, 02723F6, 02723FZ, 02723G6, 02723GZ, 02723T6, 02723TZ, 02723Z6, 0272446, 027244Z, 0272456, 027245Z, 0272466, 027246Z, 0272476, 027247Z, 02724D6, 02724DZ, 02724E6, 02724EZ, 02724F6, 02724FZ, 02724G6, 02724GZ, 02724T6, 02724TZ, 02724Z6, 0273346, 027334Z, 0273356, 027335Z, 0273366, 027336Z, 0273376, 027337Z, 02733D6, 02733DZ, 02733E6, 02733EZ, 02733F6, 02733FZ, 02733G6, 02733GZ, 02733T6, 02733TZ, 02733Z6, 02733ZZ, 0273446, 027344Z, 0273456, 027345Z, 0273466, 027346Z, 0273476, 027347Z, 02734D6, 02734DZ, 02734E6, 02734EZ, 02734F6, 02734FZ, 02734G6, 02734GZ, 02734T6, 02734TZ, 02734Z6, 02734ZZ, 02Q03ZZ, 02Q04ZZ, 02Q13ZZ, 02Q14ZZ, 02Q23ZZ, 02Q24ZZ, 02Q33ZZ, 02Q34ZZ
CABG	B202, B212, B203, B213, B223, B233, 02100Z3, 02110Z3, 02120Z3, 02100ZC, 02100ZF, 02110ZC, 02110ZF, 02120ZC, 02120ZF, 0210083, 0210093, 02100A3, 02100J3, 02100K3, 0210483, 0210493, 02104A3, 02104J3, 02104K3, 02104Z3, 0210344, 02103D4, 0210444, 02104D4, 0211083, 0211093, 02110A3, 02110J3, 02110K3, 0211483, 0211493, 02114A3, 02114J3, 02114K3, 02114Z3, 0212083, 0212093, 02120A3, 02120J3, 02120K3, 0212483, 021249302124A3, 02124J3, 02124K3, 02124Z3, 0213083, 0213093, 02130A3, 02130J3, 02130K3, 02130Z3, 0213483, 0213493, 02134A3, 02134J3, 02134K3, 02134Z3, 3617, 3615, 3616, 3612, 3613, 3614, 3691, 02C00Z6, 02C00ZZ, 02C10Z6, 02C10ZZ, 02C20Z6, 02C20ZZ, 02C30Z6, 02C30ZZ, 02JA0ZZ, 02N00ZZ, 02N10ZZ, 02N20ZZ, 02N30ZZ, 02Q00ZZ, 02Q10ZZ, 02Q20ZZ, 02Q30ZZ, 02Q33ZZ, 02Q34ZZ
Ventricular dysrhythmia	I490, 4274
Intubation	0BH17EZ, 0BH13EZ, 0BH18EZ, 9604, 9605
Restraint use	Z781, V4987
